# Effects of environment and globalization on the double and triple burdens of infection symptoms among under-five children across low-middle income countries using machine learning algorithms

**DOI:** 10.1186/s40249-025-01387-5

**Published:** 2025-11-20

**Authors:** Haile Mekonnen Fenta, A. Kofi Amegah, Aino K. Rantala, Inês Paciência, Jouni J. K. Jaakkola

**Affiliations:** 1 Center for Environmental and Respiratory Health Research (CERH), Research Unit of Population Health, University of Oulu, Oulu, Finland; 2 Biocenter Oulu, University of Oulu, Oulu, Finland; 3 Public Health Research Group, Department of Biomedical Sciences, University of Cape Coast, Cape Coast, Ghana; 4 Finnish Meteorological Institute, Helsinki, Finland; 5 Bahir Dar University, Bahir Dar, Ethiopia

**Keywords:** Demographic and health survey, Environmental predictors, Burden, Outcomes, Acute respiratory infections, Machine learning, Air pollution

## Abstract

**Background:**

Childhood infectious diseases and related symptoms, such as fever, cough, and diarrhea among children constitute the leading cause of death in low and middle-income countries (LMICs). We examined the environmental predictors of double and triple burden (D/TB) of infection symptoms among under-five children using multilevel machine learning (ML) methods.

**Methods:**

We used Demographic and Health Surveys (DHS) data from 58 LMICs between 2000 and 2023. These data were merged with cluster-level particulate matter and nitrogen dioxide from the National Aeronautics and Space Administration and country-level data on political, social, and economic globalization from the World Bank report. We applied multilevel models to screen out the most important predictors of D/TB symptoms and applied machine learning algorithms to predict these symptoms among children across LMICs. We trained and validated ML algorithms on (80, 70, and 60%) of the data and tested on the remaining (20, 30, and 40%) with 2, 5 and 10 cross-validations.

**Results:**

Of 1,546,243 children, 19.2%, 20.5% and 12.6% had fever, cough, and diarrhea, respectively; while the overall D/TB prevalence was 11.9% and 3.7%, respectively. The result revealed D/TB were associated with the location of a child, survey years, wealth index, family size, air pollutants, and environmental covariates. The estimated prevalence of both D/TB symptoms substantially varies across districts [intraclass correlation (intraclass correlation, ICC = 13.3%)] and countries (ICC = 8.8%). We found that the Random Forest gave the maximum Area Under the Curve of 94% and 99% for D/TBs for the K10 protocol and 80:20 training and testing dataset splits.

**Conclusions:**

The study found substantial variation in the prevalences of D/TB of illness among children under five and identified several environmental and sociodemographic predictors of these health outcomes. The Random Forest algorithm performed best in predicting these burdens. The study emphasized how integrating environmental and sociodemographic data with machine learning can enhance targeted interventions to reduce childhood infectious disease burdens in low- and middle-income countries.

**Graphic abstract:**

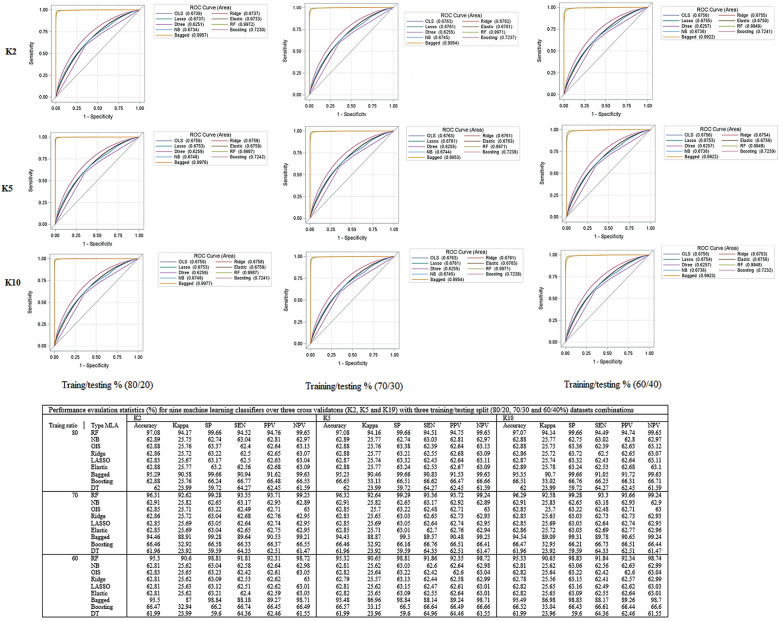

**Supplementary Information:**

The online version contains supplementary material available at 10.1186/s40249-025-01387-5.

## Background

Acute respiratory infections (ARIs) are among the most common illnesses in children, contributing to over 6% of the global disease burden, and they constitute the leading cause of death in children under five years of age [1–4]. According to the World Health Organization (WHO) in 2019, the under-five death rate due to ARIs was 73 and 9 per 1000 live births in the African and European regions, respectively, the rate in the African region being nearly eight times higher than in the European regions [3, 5]. Almost one-third of under-five years old children in low and middle-income countries (LMICs) had either pneumonia (16%); diarrhea (11%); and malaria (7%) [6]. Diarrhea [1] and fever [7] are the most common illness symptoms in under-five children, and are indicators of disorders, malnutrition, and death in children [6]. In LMICs the 2-week prevalence of fever, diarrhea, and acute respiratory infection was estimated at 18.8%, 12.5%, and 4.3%, respectively [2]. Previous literature has provided evidence that symptoms of ARIs in under-five-year-old children are directly related to environmental, socioeconomic, and cultural factors in the population of interest [4, 8–12]. Air pollution disproportionately affects the under-five children residing in LMICs, nearly 89% of deaths due to air pollution occurred in these countries, mainly in Africa and Asia [13]. It is confirmed that 92% of the world's population live in areas where the air quality index (AQI) limit is exceeded (> 100, AQI near 100 is usually considered safe) [14] and air pollution causes about 4.2 million excess deaths every year from several diseases.

Previous studies attempted to identify the determinants of fever, cough, and ARIs [4, 8–13, 15], and diarrhea [1, 6] among under-five children. However, most of these studies were focused on the determinants of these symptoms separately and in addition, they focused on Africa and Asia in one cross-sectional survey independently. Besides, authors did not elaborate the possible co-occurrence of these symptoms and considered only the classical models to identify the determinants of these symptoms. Moreover, there are no studies to date that have applied machine learning (ML) algorithms to investigate the effects of air pollutants [such as Particulate Matter (PM_2.5_), nitrogen dioxide (NO_2_)], climate factors (temperature, land surface temperature, wet day), health-related information and socio-demographic factors on double and triple burden of infections among under-five children across the LMICs over time. Furthermore, a generic prediction framework is lacking for reliable assessment of D/TB symptoms among children under five years using a large-scale dataset employing ML algorithms. To the best of our knowledge, this study employs different ML techniques to select and identify the predictors of double and triple symptoms of infections in LMICs. The predicted values of these symptoms from the best-identified MLA based on the selected features can provide essential information to help public health decision-makers to allocate limited resources and implement programs in the areas that need more attention across LMI countries. The main aim of the study is twofold: (1) to select the important risk factors of both double and triple burdens of symptoms among under-five children across the LMICs perspectives over time; (2) to compare and assess the effectiveness of various MLAs in predicting both double and triple burdens of symptoms among under-five children across the LMI countries using a diverse large dataset. Moreover, this paper leverages these large Demographic and Health survey (DHS), National Aeronautics and Space Administration (NASA) and globalization index datasets to determine the predictors of DBs and TBs among under-five children across the 58 LMICs over the year 2000 and 2023.

## Methods

### Data sources and variables

The data used in this study was obtained from different sources: the first source is the DHS https://dhsprogram.com collected from LMICs (Fig. S1 and Fig. S2). The second data source was: the National Aeronautics and Space Administration (NASA) [16]**,** where the air pollutants, such as the annual surface levels of PM_2.5_ (Fig. S3) and NO_2_ were extracted. The global and annual mean concentrations of ambient PM_2.5_ and NO_2_ [16] were the main exposure variables, and these variables were matched temporarily with the calendar year in which the DHS surveys were performed in the given countries. The third source of datasets was the Konjunkturforschungsstelle (KOF) Globalization Index which was used as a measure of globalization from 43 indicators (variables) at the country level over time (Fig. S4). The sources of the datasets, namely the Demographic and Health Surveys (DHS), NASA satellite-derived air pollution variables (PM2.5 and NO₂), and the KOF globalization indices along with the methods used to integrate these data sources, are comprehensively summarized in the conceptual framework (Fig. S5, Table S1). Detail description is of the datasets how they were matched temporarily with the calendar year in which the DHS surveys and how they managed is given in appendix (pp 2–3). Moreover, the variables included in the study, along with their descriptions, are summarized in Table S1.

*Health outcomes* This study applied three individual health outcomes, fever, cough, and diarrhea and their combinations appendix (p-7).

*Predictors* the independent variables extracted were based on a review of the literature [5, 8, 9, 11, 17–19] appendix (p-7).

### Statistical analysis

The multilevel model allows us to include the error terms at each level, that makes it possible to track changes in variance at each level across the models [20, 21]. The specific form of our multilevel regression models is, a three-level model (level 1 = children, level 2 = districts, and level 3 = country) having dichotomous outcome variables (children having the double and triple burden of symptoms of infectious diseases) ${y}_{ijk}\sim Bernoulli \left({\pi }_{ijk}\right)$, then the logit link function is:

$$log[{Pr(y}_{ijk}=1)/1-{Pr(y}_{ijk}=1]={\eta }_{ijk}={\beta }_{0}+{{{\varvec{\beta}}}_{1}{\varvec{X}}}_{1ijk}+{{{\varvec{\beta}}}_{2}{\varvec{X}}}_{2jk}+{{{\varvec{\beta}}}_{3}{\varvec{X}}}_{3k}+{v}_{k}+{u}_{jk}+{e}_{ijk}$$

$${v}_{k}\sim N\left(0, {\sigma }_{v}^{2}\right),{u}_{jk}\sim N\left(0, {\sigma }_{u}^{2}\right), {e}_{ijk}\sim N\left(0, {\sigma }_{e}^{2}\right)$$

where, ${\eta }_{ijk}=ln\left(\frac{{\pi }_{ijk}}{1-{\pi }_{ijk}}\right){\pi }_{ijk}$ is the probability that the observed DBs and TBs outcomes for U5C ${i}^{th}$ child $(i=1, . . .,\text{1,546,243})$ in ${j}^{th}$ districts $(j=1, . . .,625)$ and in the ${k}^{th}$ country$k (k=1, . . .,58)$, ${\beta }_{0}$ is the mean value across all countries, ${v}_{k}$ is the random effect of the country$k$, ${u}_{jk}$ is the random effect of districts$j$, and ${e}_{ijk}$ is the random effect of child-level residual error terms. The BLUPs (i.e., predicted random effects) in multilevel models represent deviations from a group-level mean or intercept, and these deviations can be either positive or negative [22, 23]. Thus, the expanded variance term allows us to account for the variance arising at child, district, and country levels. Moreover, ${{\varvec{\beta}}}_{1},{{\varvec{\beta}}}_{2}$ and ${{\varvec{\beta}}}_{3}$ are the vectors of fixed effect parameters for child-level, district-level, and country level covariates respectively. For the model, the adjusted odds ratios (OR) and the corresponding 95% confidence intervals (CIs) were estimated.

The intraclass correlation (ICC): the ICC was computed to assess the district and country effect/variability. It reveals the variation in the DB and TB explained by the districts and countries and computed as follows [24, 25]:

$$ ICC_{districts} \, = \frac{{\sigma_{districts}^{2} }}{{\sigma_{districts}^{2} + \sigma_{country}^{2} \, + {\raise0.7ex\hbox{${\pi^{2} }$} \!\mathord{\left/ {\vphantom {{\pi^{2} } 3}}\right.\kern-0pt} \!\lower0.7ex\hbox{$3$}}}},ICC\,attributable\,to\,level\,2{ } $$

$$ ICC_{country} \, = \frac{{\sigma_{country}^{2} }}{{\sigma_{district}^{2} + \sigma_{country}^{2} + {\raise0.7ex\hbox{${\pi^{2} }$} \!\mathord{\left/ {\vphantom {{\pi^{2} } 3}}\right.\kern-0pt} \!\lower0.7ex\hbox{$3$}}}},{\text{ ICC}}\,attributable\,to{\text{ level 3}} $$

### Machine learning algorithms

From the multilevel model results, the important risk factors (predictors) for DBs and TBs were selected. All the risk factors were selected using the threshold *P*-value of < 0.05. One of the challenges of the ML approach is the imbalanced data problems [25] that the category of one class label exceeds the other label in significant size. Under-sampling is a method in which samples from the majority group are randomly chosen without replacement until the label’s balance is attained [26], while oversampling is a technique in which samples from the minority group are randomly chosen with replacement and added to the training dataset and as a result ML based classifier is performance is enhanced [27, 28]. In our dataset, the DBs (TBs) outcome classes are significantly imbalanced, with 1,319,571 (1,443,779) samples in the “No” classes and only 179,924 (55,716) samples in the “Yes” classes respectively. As a result, unless the data is balanced, the trained MLA-based system favors the majority class when classifying the imbalanced datasets [29], more likely to categorize new observations as having no DBs and TBs. The ratio of individuals who had no DBs and TBs is 7.33 and 25.9 respectively. This study took 4.16 times the “yes” class (4.16 × 179,924 = 748,484.84) children having DBs using oversampling and the remaining 751,011 under-five children who did not have DBs from 1,319,571 using the under-sampling technique to minimize the disparities between each category. Similarly, this study took 13.4 times the “yes” class $\left(13.40 \times \text{55,716}=\text{746,594}\right)$ children having TBs using oversampling and the remaining 752,901 under-five children who did not have TBs from 1,443,779 using the under-sampling technique to minimize the disparities between each category.

### Data preprocessing and partitioning

**Fig. 1:**
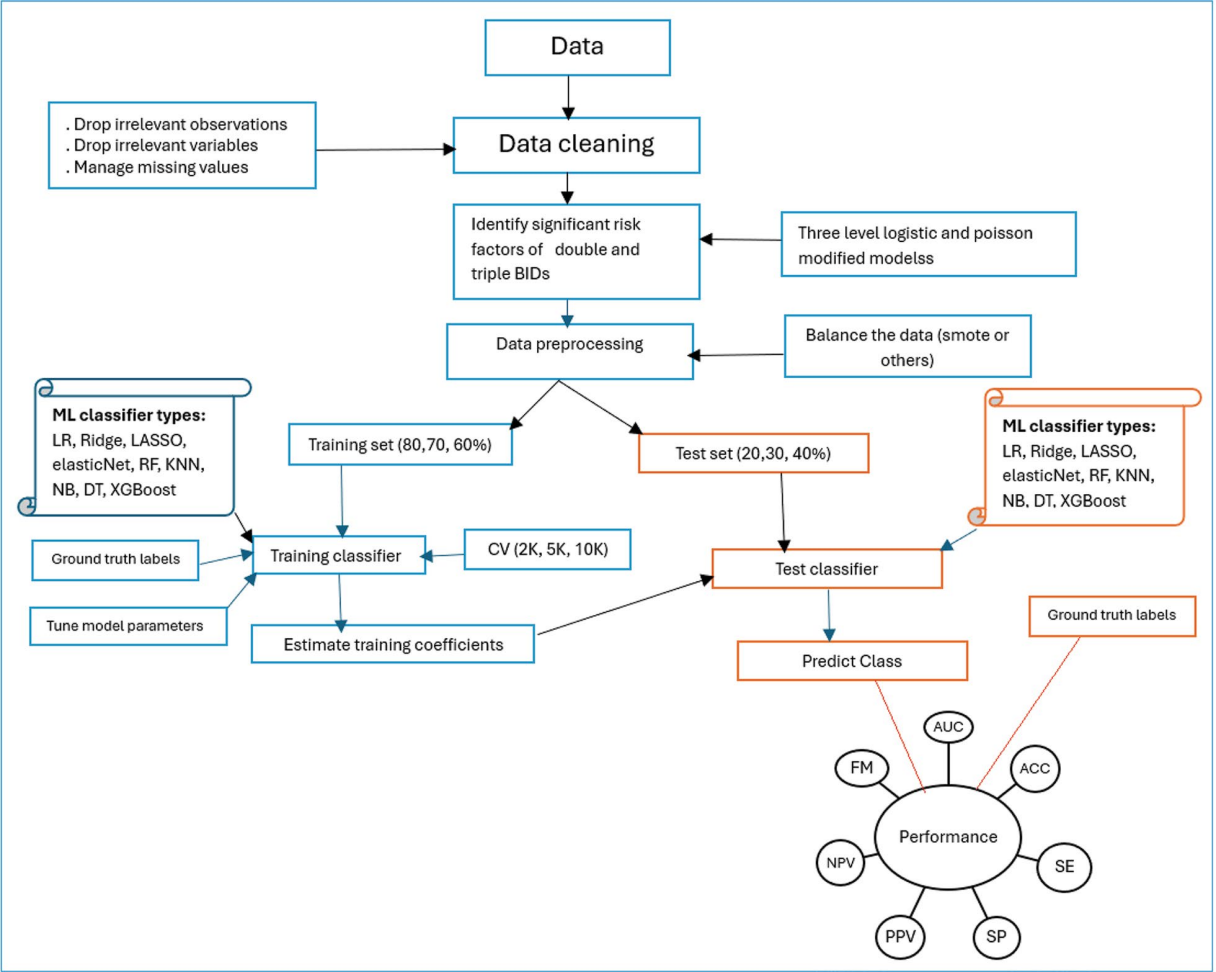
Overview flow chart of the machine learning algorithms used for predicting double burden of infectious diseases (DBs) and double burden of infectious diseases (TBs) among under-five children. *CV* cross-validation, *SE* sensitivity, *SP* Specificity, *PPV* positive predictive value, *NPV* negative predictive values, *FM* F-measure, *ACC* Accuracy, *BID* burden of infectious diseases, *LR* logistic regression, *LASSO,* *RF* random forest, *KNN* K-Nearest Neighbors, Elastic Net, *NB* Naïve Bayes, *DT* decision tree

This process repeated with (7∶3) and (6∶4) split and then 2, 5, and tenfold [2 K, 5 K, and 10 K] cross-validation was used to assess the impact of different training and testing ratios on the performance of the different machine learning algorithms (MLA) [30–35]. To select suitable MLA, we reviewed related works using ML algorithms on different childhood health outcomes such as childhood nutrition status, anemia status, and mortality [36–41]. For this study, different ML algorithms such as generalized linear models (binary logistic regression [42], Ridge [43], Least Absolute Shrinkage and Selection Operator (LASSO) regression [44] and Elastic Net [33, 45]), Random Forest (RF) [33, 46], Naïve Bayes [47–49] and Decision Trees [49] were adopted for predicting the DBs and TBs status of children aged under five years residing in 58 LMICs (Fig. 1). The study used the most recent DHS datasets (*n* = 689,146 under-five children) to validate the suggested approach.

### Performance measures and model validation

Model sensitivity and specificity relationships are expressed using receiver operating characteristics (ROC) curves, which are calculated based on the true and predicted outcome of interest. All the curves that are plotted to the left of the diagonal line are performing better than chance. The AUC gives an aggregated value which explains the probability that a random sample would be correctly classified by each of the ML algorithms [31, 50]. The identified best-fit model is then used to predict the health outcome status in another dataset, known as the test dataset [30–35] (Fig. 1).

## Results

**Fig. 2:**
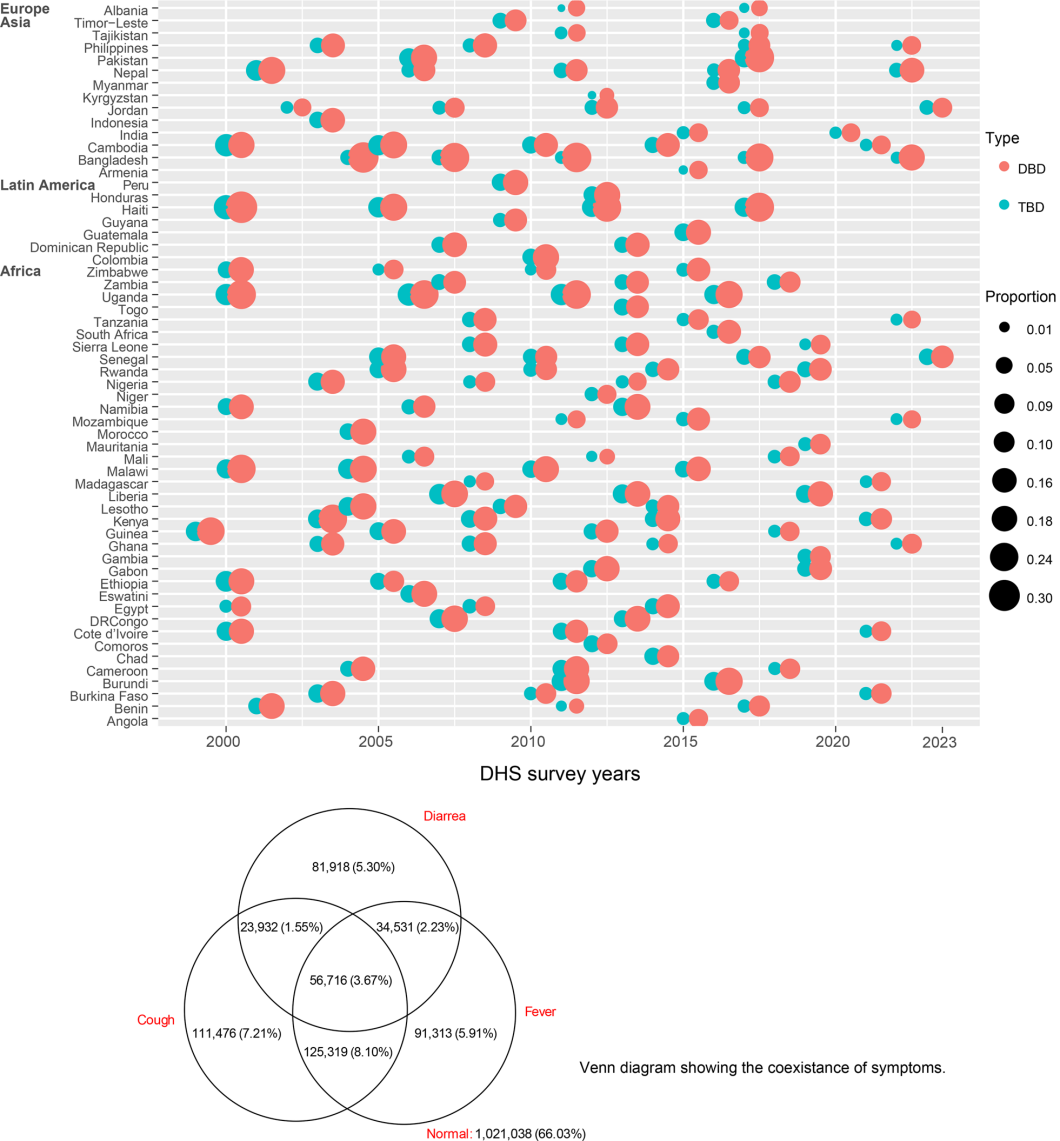
Co-existence of symptoms among children under five years across LMICs from 2000 to 2023

Overall, there was a substantial variation in the occurrence of double and triple burden of infection symptoms among under-five children across 58 LMICs for the past two decades (2000‒2023). However, the size of the point over time decreased implying that the prevalence of those symptoms slightly improved over time (Fig. 2). Bangladesh (Asia), Haiti (Latin America) and Uganda (Africa) consistently showed the highest prevalence of both double and triple infections over time. The Venn diagram revealed that nearly two-thirds (66%) of 1,021,038 under-five children were free from any of the symptoms (fever, cough, and diarrhea), while 183,143 (11.80%) experienced a double burden of infection symptoms. Moreover, 317,443 (20.53%), 307,879 (19.91%), and 197,097 (12.80%) children had cough, fever and diarrhea respectively (Fig. 2).

The co-occurrence of symptoms specifically diarrhea and cough, cough and fever, and diarrhea and fever among children under five years of age across 58 countries is summarized in Fig. S6. This figure highlights significant variation in the patterns of symptom co-occurrence between countries. Moreover, the distribution of double and triple burden of symptoms among under-five children varies across the 625 administrative districts and detailed explanation is given in appendix (p–9) and Fig. S7.

The prevalence and predictors of DBs and TBs symptoms among children (*n* = 1,546,243) under-five years across 58 LMICs are summarized in Table S2. Across all country-years, 183,782 (11.89%) and 56,716 (3.67%) of under-five children had double burden and triple burden of infection symptoms respectively. More than half (50.42%) of the sampled children were from Africa. Over one-fifth (20.14%) of children from Latin America, 13.05% from Africa, and 9.75% from Asia had the double burden of symptoms, while 4.31%, 6.64%, and 2.65% of children residing in Africa, Latin America and Asia had the triple burden of infection symptoms, respectively. Overall, 26,362 (19.24%) and 9620 (7.02%) children from the first DHS survey year (1999–2004), and 26,140 (8.04%) and 6184 (1.90%) from the recent phases (2020–2023) were experiencing DB and TBs, respectively. Nearly 72% of them resided in rural areas, and approximately three-fourths of the respondents had more than four family members (75.42%). Nearly 23%, 35%, and 31% of children came from a household with unclean fuel use for cooking, poor sanitation conditions, and households where drinking water is untreated. Almost half (47.30%) of children came from households with high household smoking risks, almost 99% of them resided in residence areas exposed to higher than WHO recommended PM_2.5_ level (above 5 µg/m^3^) and 6.31% were exposed to higher than WHO recommended NO_2_ (10 µg/m^3^) level. We present detailed description of the presults in the appendix (pp 18 − 19) and Table S2.

**Fig. 3:**
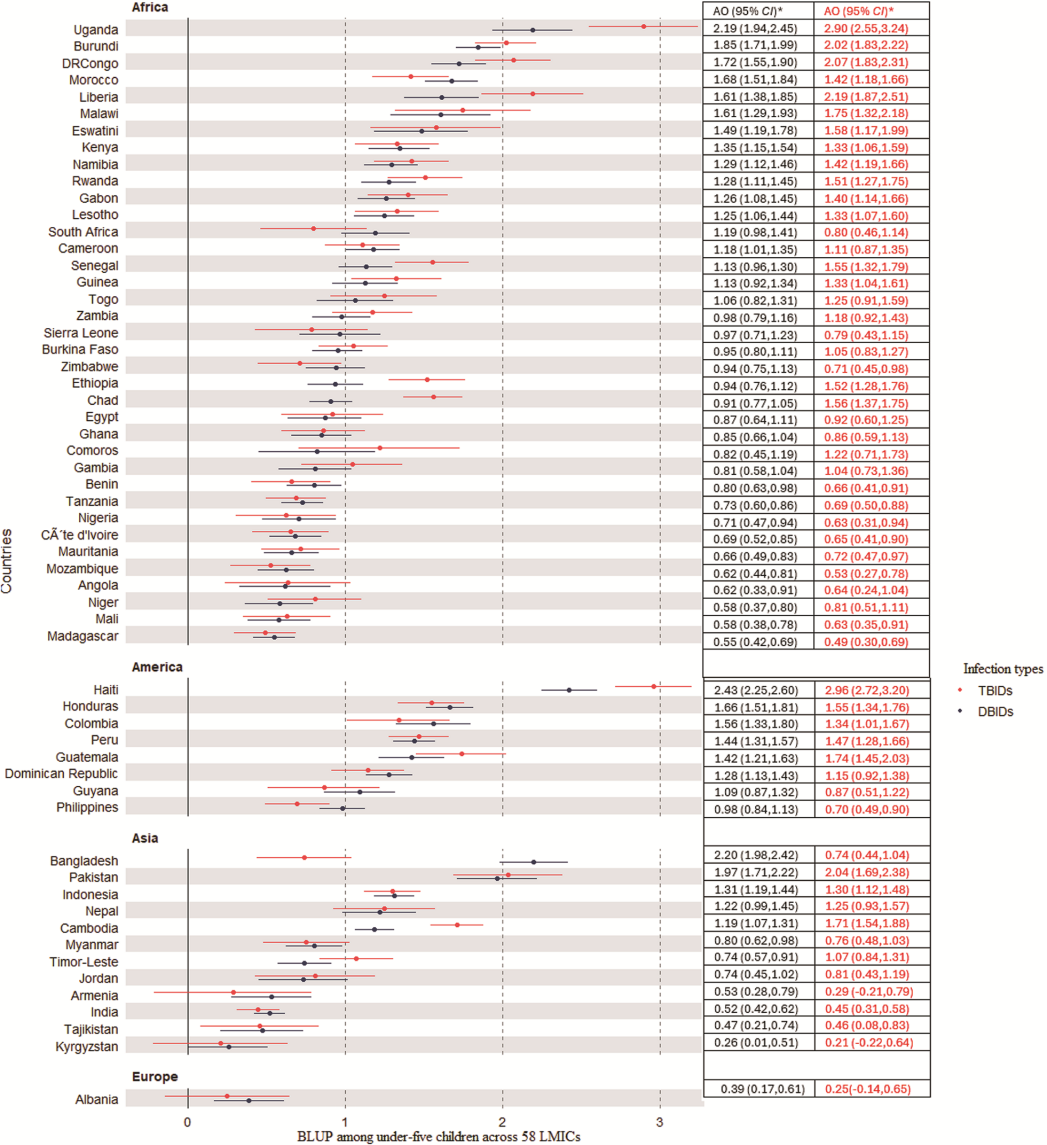
The adjusted log odds (AO) on the best linear unbiased prediction (BLUP) of burdens of symptoms among under-five children across 58 low-middle income countries (LMICs). *TBIDs* Triple Burdens of Infection disease, *DBIDs* Double Burdens of Infection diseases

Figure 3 illustrated the country-level variances by showing the average country-level intercepts from the multilevel model for both D/TB symptoms of infections. The BLUP results indicated that the value above zero (group average) reveal higher log-odds of both DB/TB symptoms, while the average and the negative value indicates lower odds. The result revealed that Uganda, Burundi, and the Democratic Republic of Congo from Africa have the highest predicted values of both double and triple burden of symptoms. Specifically, Uganda has 2.19 and 2.90 higher log-odds of DB/TB infections among under-five children compared to the average infection levels among children residing in 58 LMICs. However, Madagascar, Mali, and Niger have the lowest predicted values of infection symptoms among children in the continent. From Latin America, Haiti has the highest predicted values of both double and triple burdens while the Philippines has the lowest odds of having both double and triple burdens. From an Asian context, Bangladesh and Pakistan have the highest predicted values of both double and triple burden of symptoms while Kyrgyzstan and Tajikistan have the lowest burden.

To develop a predictive model for both DB and TB (Fig. S8) symptoms, nine ML models with three protocols (K_2_, K_5,_ and K_10_), with three different combinations of training and testing dataset splits (80∶20, 70:30 and 60:40%) are summarized in Fig. S8 and detail explanation is included in the appendix (p 3).

## Discussion

We used a large dataset of over 1.5 million under-five children in 58 LMICs from Africa, Latin America, Asia, and Europe (Albania)to identify the risk factors and predict the double and triple burden of infections among children. This study demonstrates the use of multilevel models to identify the risk factors of both double and triple-burden symptoms among under-five children and implement the ML approaches for predicting these outcomes across 58 LMICs over time. The study utilized the dataset from DHS, NASA and the globalization index from WHO reports to examine the link between the environment and the coexistence of symptoms of infections (i.e., fever, cough, and diarrhea) in a global context, including Africa, Asia, and Latin America. Results revealed a significant geographical variation in the occurrence of both double and triple burden of infections among under-five children with a substantial burden of childhood symptoms being present in Latin America. Moreover, the study highlighted a large variation in country-level and district prevalence and predicted values of these symptoms among under-five children. Previous literature revealed that the distribution of the prevalence of acute respiratory symptoms varies from country to country [8–10, 18] and from district to district within the same country [9, 12, 18, 51]. A child who resided in Latin America had a higher risk of both DB and TB compared to those who resided in Asia and Africa. This might be the reason that in Latin America, due to rapid urbanization and industrialization, many of the areas are affected by pollutant emissions [52–55].

A previous study comparing the prevalence of respiratory illnesses between the U.S. and Latin America showed that the Latin American region has a higher occurrence of respiratory conditions, such as asthma [56]. The predicted odds ratio and prevalence of cough, fever and diarrhea symptoms in Haiti, Bangladesh, Cambodia, Pakistan, the Democratic Republic of Congo, Malawi, and Uganda contribute significantly to the highest prevalence of both symptoms, this might be the reason that these countries are characterized as having relatively unstable political situations in the time of data collection. Political instability often leads to weakened infrastructure, healthcare systems, and vaccination programs, and reduces access to improved water and sanitation, which significantly increases the vulnerability to infection symptoms [57–61]

Children’s exposure to higher household smoking is positively associated with a high prevalence of both double and triple symptoms, which is in line with previous studies [62, 63]. This result also supports research [2, 6, 9, 12, 64] revealing that a higher risk of both double and triple burden of infections is significantly associated with household poverty.

In recent years, with the availability of large health-related data repositories and advances in computing power, classical statistical analysis is being combined with advanced machine learning algorithms to predict and classify the target variables [65–67]. As compared to other conventional statistical methods, the ML techniques revealed superior predictive capabilities in both outcomes among children. This is not a surprise result, as ML algorithms have been shown to outperform traditional statistical techniques in several fields [28, 37, 41, 68–72]. Moreover, the random forest (RF) outperformed better than the other algorithms in predicting both double and triple infections among children in LMICs. As also revealed in previous literature, the RF techniques have a better capability of predicting binary outcomes including mortality rates [28, 38, 73], acute respiratory infection [41], undernutrition status among children [37]. However, the performance of different ML techniques may vary depending on the nature of the dataset and the specific problem to be addressed and hence it is recommended to evaluate their performance based on the dataset we have in hand. Even though, there is literature on the risk factors of diarrhea, fever, and cough [1, 2, 6, 9, 10, 12], the prediction of both double and triple symptoms of infections using both multi-country and multi-sources datasets among under-five children across LMICs has not been explored.

### Biological plausibility

Several mechanisms may be relevant for explaining the observed association between ambient PM2.5 and the outcome symptoms. Our results suggest that those children who are exposed to higher levels of PM2.5, namely levels above the WHO recommendation, have a higher risk of having DBIDs and TBIDs. The relationship between exposure to PM2.5 and fever is primarily mediated through the activation of the immune system, oxidative stress, and systemic inflammation. Exposure to ambient PM2.5 may increase the inflammatory response and the release of pro-inflammatory cytokines such as interleukin-1 (IL-1), IL-6, and C-reactive protein (CRP), which are key mediators in the development of fever [74–76] These cytokines may also act on the hypothalamus increasing the production of prostaglandin E2, a principal mediator of fever [74, 77, 78]. In addition to promoting inflammation, exposure to PM2.5 may induce oxidative stress, which further amplifies cytokine (IL-6 and TNF-α) production and inflammatory signaling pathways, and consequently contributing to fever [79, 80]. Exposure to PM2.5 has also been associated with coughing through both direct and indirect pathways involving inflammation, oxidative stress, and neural activation. Exposure to PM2.5 was associated with an upregulation of inflammatory pathways, such as the NF-kB signaling pathway, leading to increased production of proinflammatory cytokines (*i.e.*, IL-1β, IL-6, and TNF-α), and consequently contributing to airway inflammation and cough [81]. PM2.5 may also increase the expression of transient receptor potential vanilloid-1 (TRPV1) in the airway epithelium, leading to increased cough symptoms [82–84]. Additionally, TRPV1 activation by PM2.5 may result in increased levels of substance P, a neuropeptide associated with inflammation and cough reflex sensitivity [83]. Similar to fever, the oxidative stress associated to PM2.5 may activate kinase cascades and transcription factors, releasing inflammatory mediators that contribute to airway inflammation and cough [85, 86]. Our results also highlight the association between exposure to PM2.5 and diarrhea, which may be supported by several interrelated mechanisms involving gut barrier dysfunction, inflammation, oxidative stress, and alterations in the gut microbiota. Inhaled PM2.5 can enter the gastrointestinal tract through mucociliary clearance or direct ingestion [87]. Once in the intestine, PM2.5 can increase epithelial permeability and impair the function of the intestinal immune barrier, leading to intestinal injury and inflammation that may contribute to gastrointestinal complications such as diarrhea [88, 89]. Additionally, PM2.5 exposure has been shown to induce intestinal inflammation by disrupting the balance of regulatory T cells (T reg) and T helper cells (Th17) and increasing the release of pro-inflammatory cytokines like IL-6, IL-1β, and TNF-α, which are crucial for maintaining intestinal immune homeostasis, increasing the risk of diarrhea [89–92]. Several studies have provide evidence on the adverse effects of exposure to PM2.5 and gut microbiome [92–95] According to scholars [90] showed a significant decrease of *Lactobacillus acidophilus* after exposure to PM2.5, which has been associated with an imbalance of Treg/Th17. The review conducted by [94]including 12 studies on humans, showed that exposure to air pollution exposure was positively associated with bacterial taxa belonging to Bacteroidetes, Deferribacterota, and Proteobacteria, and negatively associated with bacterial taxa belonging to Verrucomicrobiota, this observed gut dysbiosis may impair digestion and immune regulation, leading to gastrointestinal disturbances including diarrhea[96].

The study population comprised over 1.5 million children which provided accurate estimates of the prevalences of the studied health outcomes. The sampling techniques used ensure representativeness of the prevalence and measures of effect across wide geographical areas including 58 countries. The algorithms were rigorously tested on this comprehensive dataset and machine learning techniques were utilized to predict the double and triple coexistence of infections among children under five years. The potential predictors were incorporated from a wide range of data sources from various perspectives, including globalization index (political, social, and economic), NASA and DHS, to ensure a robust and multifaceted analysis.

The main limitation of the study is the cross-sectional design, which does not allow elaboration of temporality between hypothesized determinants and predictors and the health outcomes of interest. This limits any causal inference to be made from the results. Another limitation is that the study (survey) was conducted in different years, and the comparison made on prevalence by country may be bias because of the changes in determinants and health outcomes over time. Finally, information on the health outcomes (fever, cough, and diarrhea) and some hypothesized determinants and predictors of these outcomes were self-reported by mothers/caregivers, and as a result, maternal recall bias may have affected the validity of the studied determinant-outcome relations.

## Conclusions

Our study used PM_2.5_ from NASA and the globalization index from WHO reports to assess the association between the environmental pollution and globalization index with both double and triple burden of infections among 1,546,243 under-five children sampled from the 2000 to 2023 DHS dataset in 58 LMICs. The study explores a full statistical analysis and machine learning algorithms of variables associated with both double and triple burden of infection symptoms among under-five children resided in LMICs, employing multilevel models and machine learning algorithms. The multilevel model revealed that the location of a child, survey years, wealth index of the households, residence, size of the household PM_2.5_, fuel use, sanitation, sources of drinking water, and level of smoking risk were some of the significant factors of both double and triple burden of infections.

The present study attempted to identify the best ML algorithms for the prediction of both double and triple symptoms of infections using nationwide cross-sectional data from 58 LMI countries. The analysis of different MLA for both double and triple symptoms of infection prediction has shown that the best-performing algorithms are RF and bagged trees. Their consistently outstanding performance was checked across multiple evaluation metrics with three different protocols and three different training–testing ratios leading to this conclusion. The future direction of our research would be to explore the longitudinal analysis that could track the temporal trends and progress at double and triple rates over time, offering insights into the child public health.

## Supplementary Information

Supplementary file 1.

## Data Availability

The datasets used in this study are publicly available and can be accessed from portals. The DHS data are publicly available from https://dhsprogram.com after a formal request is accepted, while the PM_2.5_ and NO_2_ estimates are publicly available as version V4.GL.03 at https://sites.wustl.edu/acag/datasets. Moreover, the KOF Globalization Index (KOF Globalisation Index – KOF Swiss Economic Institute | ETH Zurich) and the World Bank (https://data.worldbank.org/). The SAS, STATA and R codes used in the study will be available from a formal request from the corresponding author.

## Abbreviations

ARIs: Acute respiratory infections
LMICs: Low and middle-income countries
D/TB: Double and triple burden
DHS: Demographic and health surveys
MLA: Machine learning algorithms
ICC: Intraclass correlation
ACU: Area under the curve
WHO: World Health Organization
PM_2.5_: Particulate matter
NO_2_: Nitrogen dioxide
NASA: National aeronautics and space administration
ROC: Receiver operating characteristics
KR: Kids record
SER: Smoke exposure risks
GPS: Global positioning system
PCA: Principal component analysis
