# Supplementary material for: Effects of environment and globalization on the double and triple burdens of infection symptoms among under-five children across low-middle income countries using machine learning algorithms
Source: Infect Dis Poverty. 2025 Nov 20;14:117. doi: 10.1186/s40249-025-01387-5 (PMC12632089; doi:10.1186/s40249-025-01387-5)
Supplement: Supplementary file 1 [file 40249_2025_1387_MOESM1_ESM.docx]

**Appendix**

[**Supplementary Material S1: Data sources and variables** 2](#_Toc205544155)

[**Supplementary Material S2: Results** 8](#_Toc205544156)

# **Supplementary Material S1: Data sources and variables**

The DHS program conducts nationally representative surveys from women aged 15-49 years, and their children born in the last five years before the survey in more than 90 LMICs. There are more than 260 DHS surveys in over 90 countries advancing the global understanding of population and health trends, especially in developing countries. Children’s files (kids Record-KR files) were used for our analysis, which includes the datasets that were collected from the years 2000 to 2023 at different points in time in the given countries. Different types of household cooking places (outdoor and indoor cooking places and cooking fuels (non-smoke-producing and smoke-producing cooking fuel) were used from household members' (PR-records) record files and the household smoke-exposure risks (SERs) were computed [1]. After relevant inclusion and exclusion criteria (surveys restricted or non-available, surveys with no GPS locations, and the like), our final analysis includes 146 surveys from 58 LMICs in the period 2000-2023 (Fig. S1). The number of surveys for each country varies and ranges from a minimum of one (Armenia, Angola, Myanmar, and Peru) to a maximum of five (Bangladesh, Nepal, and Jordan). This is a rich dataset providing insights into DHS over two decades across diverse countries on different continents. An overview of the countries included in the study, the sample size and survey years are shown in (Fig. S2). The National Aeronautics and Space Administration (NASA) [2]**,** where the air pollutants, such as the annual surface levels of PM_2.5_ (Fig. S3) and NO_2_ were extracted. The DHS dataset has a separate shape file of the clusters (Enumeration areas) which consists of the cluster IDs with their GPS locations (Longitude, Latitude). The global and annual mean concentrations of ambient PM_2.5_ and NO_2_ [2] were the main exposure variables, and these variables were matched temporarily with the calendar year in which the DHS surveys were performed in the given countries. Those air pollutant variables were extracted from the raster images (GeoTIFF) using the open-source R software via the GPS locations (longitude and latitude) which were extracted from the DHS shape files. Children within a given cluster (enumeration areas) were assigned to have the corresponding exposure (PM_2.5_ and NO_2_) estimates. The KOF is defined across three distinct dimensions such as political, social, and economic globalization (<http://globalization.kof.ethz.ch/>), and each of the dimensions is aggregated from different subcomponents. The KOF values range from 0 to 100, with lower values revealing lower levels of globalization and the data is available for 215 countries worldwide between 1970 and 2021 [3]. These three variables (economic, social, and political globalization indices) were linked to the original DHS datasets with country and year as a common key variable and almost in all countries (Fig. S4), which increased over time. The variables included in the study, along with their descriptions, are summarized in Table S1.

| Table S1: Variables included in the study and their descriptions | | | | |
| --- | --- | --- | --- | --- |
| Variable | Coding / Type | Source | Level | Notes |
| Fever | Binary (1 = Yes, 0 = No) | DHS (KR File) | Individual (child) | Symptoms in the last 2 weeks |
| Cough | Binary (1 = Yes, 0 = No) | DHS (KR File) | Individual (child) | Symptoms in the last 2 weeks |
| Diarrhea | Binary (1 = Yes, 0 = No) | DHS (KR File) | Individual (child) | Symptoms in the last 2 weeks |
| Double Burden (DBs) | Binary (1 = any 2 symptoms, 0 = otherwise) | Derived | Individual (child) | Based on fever, cough, and diarrhea |
| Triple Burden (TBs) | Binary (1 = all 3 symptoms, 0 = otherwise) | Derived | Individual (child) | Based on fever, cough, and diarrhea |
| Mother's Age | Categorical (15–29, 30–34, 35–39, 40–49) | DHS | Individual (mother) | Grouped for modeling |
| Mother's Education | No education, Primary, Secondary, Higher | DHS | Individual (mother) | Reference: No education |
| Birth Type | Binary (0 = Single, 1 = Multiple) | DHS | Individual (child) | Twin/multiple birth status |
| Birth Order | 1, 2–3, ≥4 | DHS | Individual (child) | Order of child’s birth |
| Birth Interval | <18, 18–59, ≥60 months | DHS | Individual (child) | Time between births |
| Birth Weight | Binary (1 = Low, 0 = Normal) | DHS | Individual (child) | Low birth weight defined by DHS |
| Child Age | 6–11, 12–23, 24–35, 36–59 months | DHS | Individual (child) | Used to measure burden by age group |
| Child Sex | Male = 0, Female = 1 | DHS | Individual (child) | Used in model as a binary predictor |
| Place of Delivery | Binary (0 = Home, 1 = Health facility) | DHS | Individual (mother) | Delivery location |
| Cesarean Delivery | Binary (0 = No, 1 = Yes) | DHS | Individual (mother) | Mode of delivery |
| Cooking Fuel Type | Binary (1 = Unclean, 0 = Clean) | DHS PR file | Household | Based on fuel type (wood, dung vs gas, electricity) |
| Toilet Type | Binary (1 = Improved, 0 = Unimproved) | DHS | Household | Based on WHO/UNICEF classification |
| Water Source | Binary (1 = Improved, 0 = Unimproved) | DHS | Household | WHO/UNICEF definition |
| Household Smoke Exposure (SER) | Low, Medium, High | Derived from PR File | Household | Computed based on fuel + cooking place |
| Wealth Index | Quintiles (Poorest to Richest) | PCA / DHS | Household | PCA-derived for surveys before 2005 |
| Residence | Binary (1 = Urban, 0 = Rural) | DHS | Household | Urban/Rural dwelling |
| Household Size | <4, 5–9, ≥10 members | DHS | Household | Categorized household size |
| PM2.5 Exposure | Binary (1 = >5 μg/m³, 0 = ≤5 μg/m³) | NASA + DHS GPS | Cluster | Linked to DHS via GPS points |
| NO2 Exposure | Binary (1 = >10 μg/m³, 0 = ≤10 μg/m³) | NASA + DHS GPS | Cluster | Linked via satellite + cluster location |
| Cluster ID / GPS Coordinates | Latitude & Longitude | DHS Shapefiles | Cluster | For matching environmental raster data |
| DHS Phase | Phase I (1999–2004) to Phase V (2020–2023) | DHS | Country-Year | Based on year of survey |
| KOF Globalization Indices | Economic, Political, Social (0–100 scale) | KOF Globalization Index | Country-Year | Merged via country and year |
| Region | Africa, Asia, America, Europe | DHS | Country | Africa as reference |

Note; DHS: Demograpphic and Health Survey;KR: kids Record; PR: Household Member Recode; PCA:Principal component analysis; NASA:National Aeronautics and Space Administration


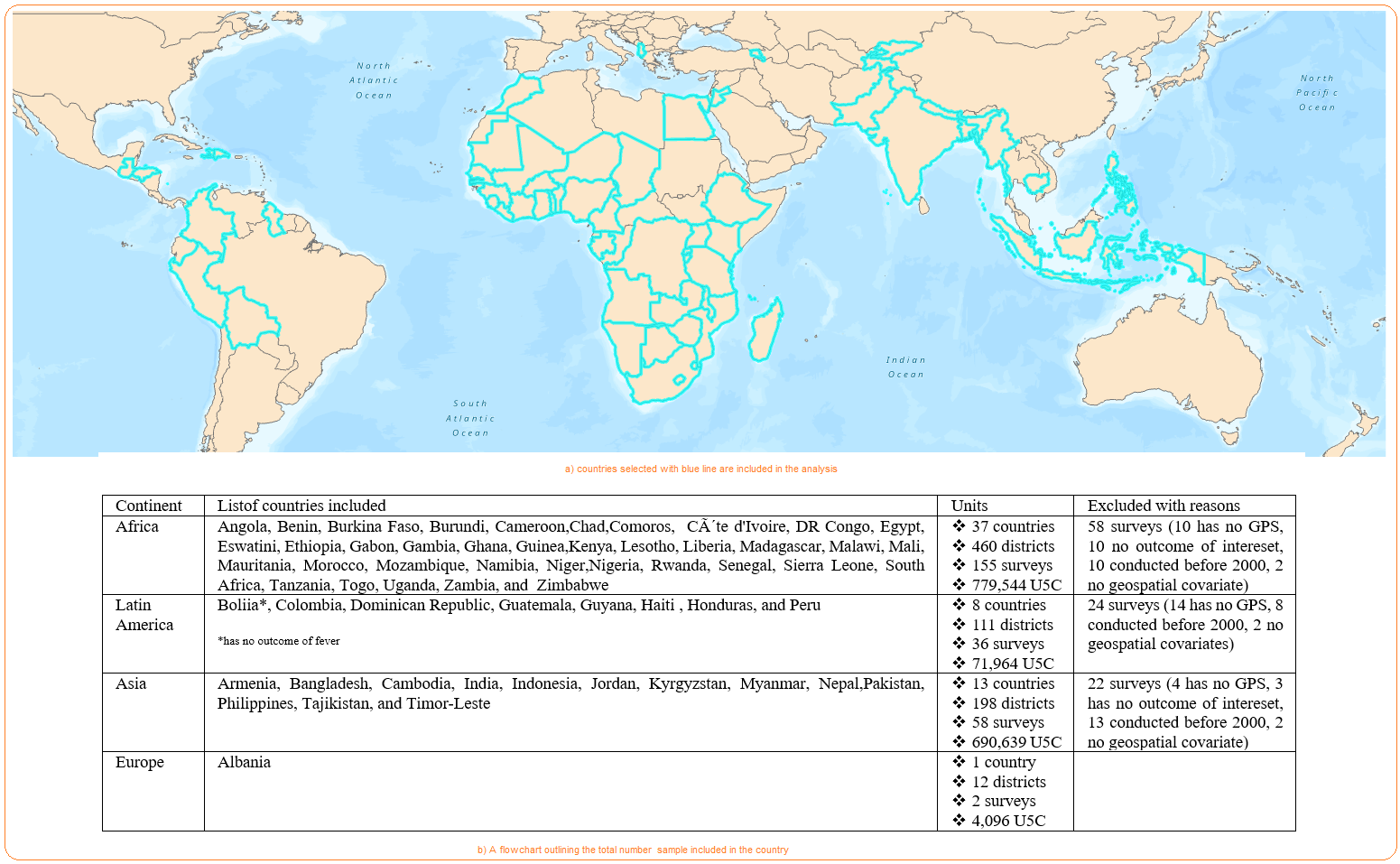


Note: DRCongo, Democratic Republic of Congo

Fig. S1: The flowchart for selection of countries and individuals included in the analysis: Blue line showing the DHS survey data collected from eligible countries, DHS data (2000-2023).

Fig. S2: Overview of the countries included in the study by survey years: the size and color of the points represent the sample size (N) of each survey and the countries, respectively.

Fig. S3: change of PM_2.5_ over time across the low-middle income countries.

Fig. S4: The three globalization index variables (economic, social, and political) with survey year over time.

This framework given in Fig. S5 illustrates how the Demographic and Health Surveys (DHS), NASA satellite-derived air pollution variables (PM2.5 and NO₂), and the KOF globalization indices are temporally linked with environmental exposures and macro level globalization indicators to enable a multidimensional analysis across low- and middle-income

countries.


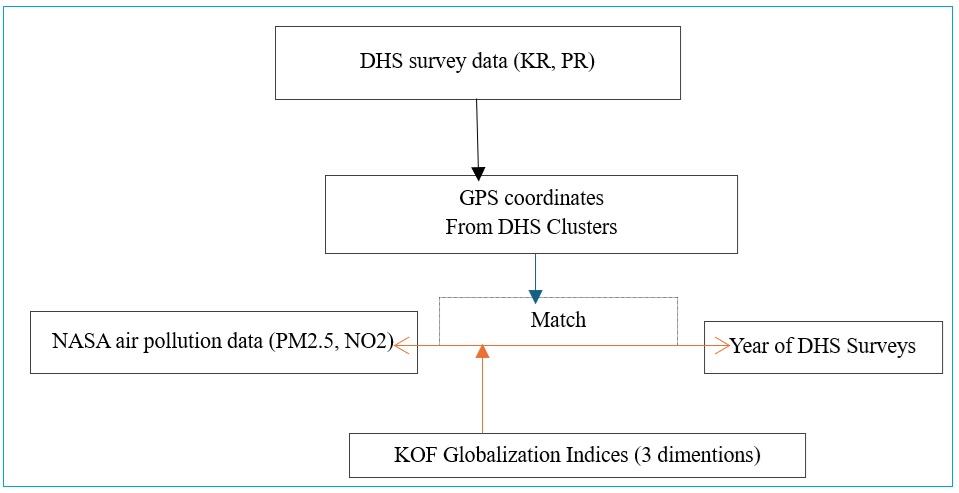


Fig. S5: Conceptual Framework Diagram for the integration of all different data sources

**Health outcomes:** To measure the symptoms of infections, mothers/caregivers were asked if their alive under-five children had experienced the symptoms (fever, cough, and diarrhea), each classified as binary outcome measures (yes, no), within two weeks prior to the DHS surveys [4]. Finally, this study focused on two additional outcomes, the double burden of symptoms of infectious diseases (DBs) and the triple burden of symptoms of infectious diseases (TBs), which were computed from symptoms (fever, diarrhea, and cough). If a child has three symptoms simultaneously, they are classified as having the TB and if they suffer from only two, they are classified to have the DB.

**Predictors (Independent variables):** The wealth index of households for the DHS data conducted before 2005 was not available directly in the survey, hence we used the principal component analysis (PCA) to generate the wealth index variable scores for urban and rural areas separately. This variable was then merged together and finally, and we divided the factor scores into quintiles [5, 6].

# **Supplementary Material S2: Results**

Fig. S6: The proportion of under-five children with double occurrence of symptoms across the continents.

Districts in Chad, Tanzania, Uganda, and Senegal had in general the highest prevalence of TBs, while districts in countries including Madagascar, Zambia, Ethiopia, and some parts of Ghana and Burkina-Faso had the lowest prevalence of TBs. The proportion of DBs and TB also varied among districts in Indonesia, Cambodia, Myanmar, and Pakistan in Asian countries (Fig. S7).


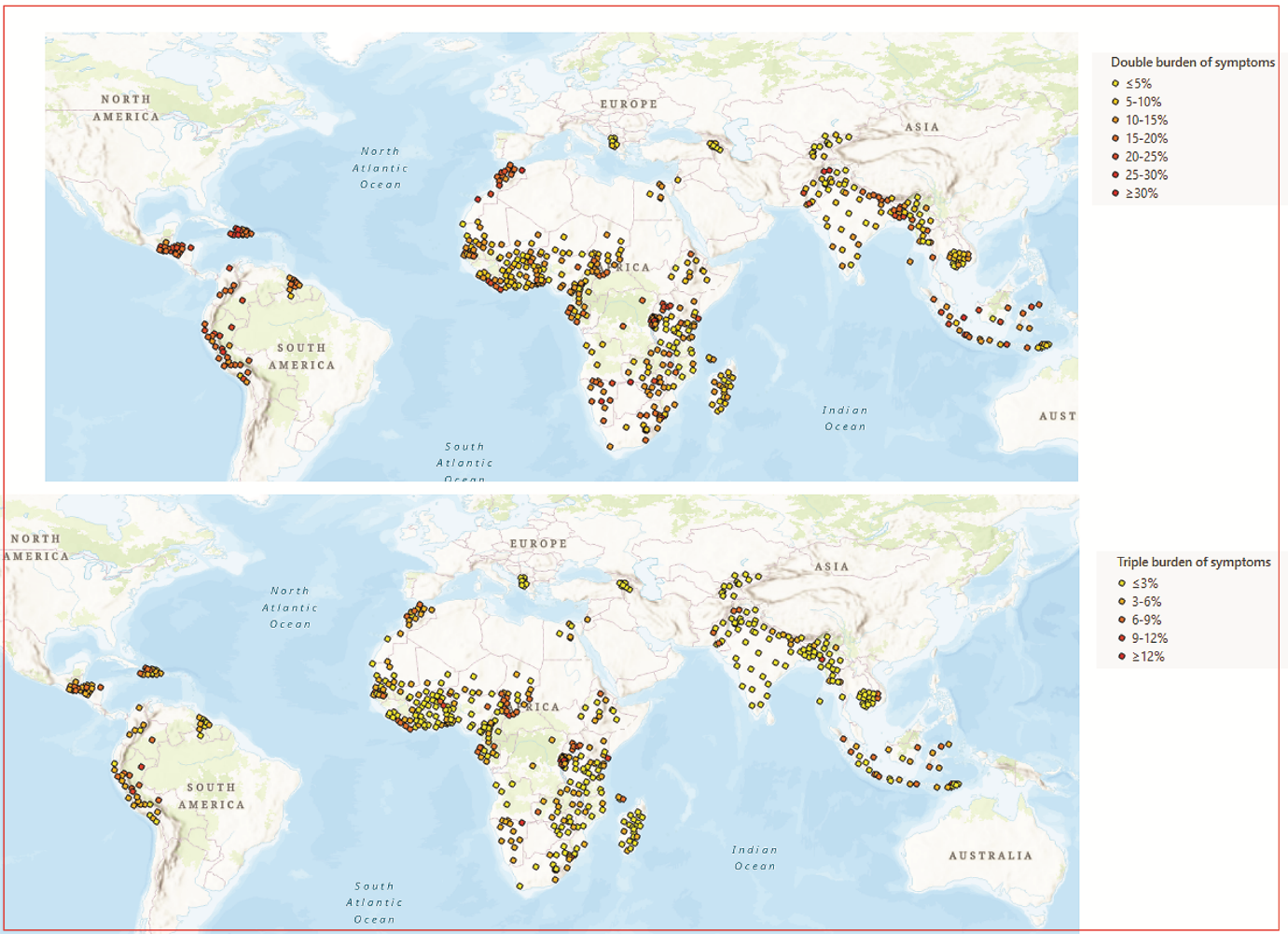


Fig. S7: Double and triple burden of infection symptoms (%) among children under five years by districts in the given LMICs

Moreover, 48,455 (12.25%) children from the poorest, 41,386 (12.22%) from the poor, 38,100 (12.4%) from the middle, 31,695 (11.3%) from the richer, and 24,146 (10.94%) from the richest households were suffering from DBs. Children from families with more than 10 members had a higher prevalence of both DBs and TBs compared with smaller family sizes (Table 2). In urban areas, 52,406 (11.77%) and 14,281 (3.21%) children had DBs and TBs, respectively. DBs and TBs were reported for 144,154 (12.35%) and 46,667 (4.00%) children when unclean fuel for cooking was used, respectively. Overall, 181,859 (11.94%) and 56,190 (3.45%) children reported DBs and TB, respectively if they were from regions with above WHO-recommended level of PM_2.5_ annual levels.

Results from the multilevel model revealed that children residing in Latin America were 45% [APR=1.45; 95% CI:1.03-2.75] and 84% [AOR=1.84; 95% CI:1.23-2.75 ] times more likely to have DB and TB symptoms compared with those residing in Africa. The odds of having TBs symptoms for children under-five in DHS phase II: IV were 0.72, 0.71, 0.68, and 0.63 respectively compared with the first phase of DHS (1999-2004). This showed that a child born in the fifth (2020-2023), and fourth (2015-2019) DHS phases were respectively 27% and 32% less likely to have TBs compared with the first phase (reference category). The odds of children having TBs symptoms in mothers aged 40-49, 35-39, and 30-34 were 1.47, 1.25, and 1.08 respectively higher compared to those born to mothers aged 15-29 years. The odds of experiencing TBs in children born from mothers who had higher education decreased by 19.40% (AOR=0.81; 95% CI: 0.96, 0.96) compared to mothers who had no formal education. Besides, the odds of having TBs of children born from households to richest and richer wealth were decreased by 31% [AOR=0.69; 95 % CI: 0.67, 0.72] and 14.40% [AOR=0.86; 95 % CI: 0.83,0.88] compared to those belonging to the poorest households, respectively. The odds of having DBs and TBs among children with low birth weight were increased by 9.10% [AOR=1.09; 95 % CI: 1.07,1.11] and 10.70% [AOR=1.11; 95 % CI: 1.08,1.12] compared with those who have normal birth weight, respectively.

Children born from households who used clean fuel for cooking and having improved toilet facilities were 16% less likely [AOR=0.84; 95 % CI: 0.81,0.87] and 13% less likely [AOR=0.86; 95 % CI: 0.83,0.88] of having TBs compared with their counter parts. Moreover, the odds of having DBs and TBs in children born in residents above the WHO recommended PM2.5 (above 5 μg/m3) level were 1.36 [95 % CI: 1.23, 1.51] and 1.77 [95 % CI: 1.44, 2.17] higher odds of those children born from below the WHO recommended PM2.5 (below 5 μg/m3) levels, respectively.

The results also revealed significant variability in DB and TB of under-five children across districts and countries, with district and country-level variances contributing to the overall variability in the experience of DBs and TBs among children across LMICs. Specifically, the districts and countries respectively accounted for 8.50% and 6% of the variations of DBs among children, while the remaining 85% of variation of DB was accounted by the under-five children. Similarly, the correlation of the presence of TBs in children from the same districts (ICC=13.30%) and from the same countries (ICC=8.80%) is not negligible.

| Table S2: Baseline characteristics overall, double and triple burden of symptoms with Adjusted Odds ratios (AOR) estimated from the multilevel model | | | | | | |
| --- | --- | --- | --- | --- | --- | --- |
| Covariates | Categories | Overall, n(%) | DBs, n (%) | AOR (95% CI | TBs, n (%) | AOR (95% CI |
| Overall prevalence |  | 1,546,243 (100) | 183,782 (11.89) |  | 56,716 (3.67) |  |
| Locations | Africa® | 779,544 (50.42) | 101,768 (13.05) | 1 | 33,627 (4.31) | 1 |
|  | America | 71,946 (4.65) | 14,494 (20.14) | 1.45 [1.01, 2.05] | 4778 (6.64) | 1.84 [1.23, 2.75] |
|  | Asia | 690,639 (44.67) | 67,308 (9.75) | 0.92 [0.67, 1.25] | 18,279 (2.65) | 1.05 [0.77, 1.43] |
|  | Europe | 4,096 (0.26) | 212 (5.18) | 0.40 [0.15, 1.12] | 32 (0.78) | 0.34 [0.12, 0.96] |
| DHS phases | 1999-2004®(I) | 137,022 (8.86) | 26,362 (19.24) | 1 | 9,620 (7.02) | 1 |
|  | 2005-2009 (II) | 201,148 (13.01) | 27,987 (13.91) | 0.87 [0.84, 0.90] | 9,649 (4.80) | 0.72 [0.682, 0.75] |
|  | 2010-2014 (III) | 361,934 (23.41) | 49,156 (13.58) | 0.95 [0.91, 0.99] | 15,543 (4.29) | 0.71 [0.658, 0.76] |
|  | 2015-2019 (IV) | 520,973 (33.69) | 54,137 (10.39) | 0.97 [0.92, 1.02] | 15,720 (3.02) | 0.68 [0.626, 0.74] |
|  | 2020-2023 (V) | 325,166 (21.03) | 26,140 (8.04) | 0.88 [0.83, 0.93] | 6184 (1.90) | 0.63 [0.562, 0.69] |
| Age of mother | 15-29® | 936,023 (60.54) | 112,687 (12.04) | 1 | 35,821 (3.83) | 1 |
|  | 30-34 | 317,464 (20.53) | 36,274 (11.43) | 0.99 [0.97, 1.01] | 10,633 (3.35) | 1.08 [1.046, 1.12] |
|  | 35-39 | 188,665 (12.20) | 22,231 (11.78) | 0.99 [0.97, 1.01] | 6579 (3.49) | 1.25 [1.188, 1.32 ] |
|  | 40-49 | 104,091 (6.73) | 12,590 (12.10) | 1.01 [0.97, 1.05] | 3683 (3.54) | 1.47 [1.363, 1.58] |
| Education_M | No education® | 503,433 (32.56) | 55,923 (11.11) | 1 | 20,165 (4.01) | 1 |
|  | Primary | 419,202 (27.11) | 60,912 (14.53) | 1.16 [1.14, 1.18] | 20,161 (4.81) | 1.09 [1.06, 1.12] |
|  | Secondary | 503,817 (32.58) | 55,853 (11.09) | 1.13 [1.11, 1.15] | 14,169 (2.81) | 0.97 [0.95, 1.00] |
|  | Higher | 119,791 (7.75) | 11,094 (9.26) | 0.98 [0.95, 1.01] | 2221 (1.81) | 0.81 [0.76, 0.85] |
| Wealth Index | Poorest® | 405,291 (26.21) | 48,455 (12.25) | 1 | 17,394 (4.29) | 1 |
|  | Poorer | 344,374 (22.27) | 41,386 (12.22) | 1.01 [0.99, 1.02] | 13,131 (3.81) | 0.93 [ 0.91, 0.96] |
|  | Medium | 311,484 (20.14) | 38,100 (12.4) | 0.99 [0.97, 1.00] | 11,591 (3.72) | 0.89 [0.87, 0.92] |
|  | Richer | 267,578 (17.31) | 31,695 (11.93) | 0.97 [0.95, 0.99] | 8796 (3.29) | 0.86 [0.83, 0.88] |
|  | Richest | 217,516 (14.516) | 24,146 (10.94) | 0.87 [0.85, 0.89] | 5804 (2.67) | 0.69 [0.67, 0.72] |
| Residence | Rural | 1,101,008 (71.21) | 131,376 (11.93) | 1 | 42,435 (3.85) | 1 |
|  | Urban® | 445,235 (28.79) | 52,406 (11.77) | 1.01 [0.99, 1.02] | 14,281 (3.21) | 0.99 [0.97, 1.02] |
| HHS | <4® | 380,055 (24.58) | 47,591 (12.52) | 1 | 14,370 (3.78) | 1 |
|  | 5-9 | 931,281 (60.23) | 107,842 (11.58) | 1.01 [0.99, 1.03] | 32,813 (3.52) | 0.98 [0.96, 1.01] |
|  | 10+ | 234,907 (15.19) | 28,349 (12.07) | 1.19 [1.13, 1.26] | 9533 (4.06) | 1.12 [1.08, 1.16] |
| Child’s sex | Male® | 789,037 (51.03) | 95,198 (12.07) | 1 | 30,017 (3.80) | 1 |
|  | Female | 757,206 (48.97) | 88,584 (11.70) | 0.96 [0.95, 0.97] | 26,699 (3.54) | 0.91 [0.89, 0.93] |
| Birth type | Single | 1,511,360 (97.74) | 179,570 (11.88) | 1 | 55,312 (3.67) | 1 |
|  | Multiple | 34,883 (2.26) | 4212 (12.07) | 1.02 [0.98, 1.05] | 1404 (4.02) | 1.13 [1.07, 1.20] |
| Birth intervl (months) | <18 | 537,512 (34.72) | 61,086 (11.36) | 1 | 18,373 (3.42) | 1 |
|  | 18-59 | 831,453 (53.70) | 100,063 (12.03) | 1.09 [1.06, 1.12] | 32,227 (3.88) | 1.15 [1.02, 1.20] |
|  | >=60 | 179,330 (11.58) | 22,879 (12.76) | 1.06 [1.04, 1.08] | 6177 (3.44) | 1.13 [1.09, 1.17] |
| Low birth weigt | No | 1,307,437 (85.12) | 159,594 (12.21) | 1 | 49,638 (3.80) | 1 |
|  | Yes | 228,757 (14.89) | 23,494 (10.27) | 1.09 [1.07, 1.11]*** | 6886 (3.01) | 1.12 [1.08, 1.14 ] |
| Birth order | 1 | 428,482 (27.71) | 48,697 (11.37) | 1 | 14,262 (3.33) | 1 |
|  | 2-3 | 623,910 (40.35) | 71,434 (11.45) | 1.09 [0.98, 1.11] | 21,154 (3.39) | 1.22 [1.10, 1.47 ] |
|  | >=4 | 493,851 (31.94) | 63,651 (12.89) | 1.29 [1.15, 1.44] | 21,300 (4.31) | 1.60 [1.33, 1.94] |
| Child’s age (months) | 6-11® | 328,232 (21.23) | 41,493 (12.64) | 1 | 15,377 (4.68) | 1 |
|  | 12-23 | 312,781 (20.23) | 47,467 (15.18) | 1.24 [1.22, 1.27] | 18,413 (5.89) | 1.37 [1.32, 1.41] |
|  | 24-35 | 302,869 (19.59) | 37,536 (12.39) | 0.91 [0.92, 0.98] | 10,891 (3.60) | 0.83 [0.78, 0.87 ] |
|  | 36-59 | 602,361 (38.96) | 57,286 (9.51) | 0.77 [0.73, 0.81] | 12,035 (2.00) | 0.52 [0.48, 0.57 ] |
| Place delivery | Home | 496,788 (32.14) | 46,332 (13.35) | 1 | 23,337 (4.70) | 1 |
|  | Health facility | 1,002,867 (64.86) | 112,987 (11.27) | 0.99 [0.99, 1.01] | 32,673 (3.26) | 1.00 [0.98, 1.03] |
| Fuel | Unclean | 332,811 (22.19) | 144,154 (12.35) | 1 | 46,667 (4.00) | 1 |
|  | Clean® | 1,167,169 (77.81) | 32,468 (9.76) | 0.95 [0.93, 0.96] | 7921 (2.38) | 0.84 [0.81, 0.87] |
|  | No | 1,391,614 (89.88) | 165,684 (11.91) | 1 | 51,943 (3.73) | 1 |
| cesarean delivery | Yes | 156,681 (10.12) | 18,344 (11.719 | 1.12 [1.10,1.14] | 4834 (3.09) | 1.01 [0.92, 1.05] |
| Water | Unimproved® | 484,473 (31.29) | 65,049 (13.43) | 1 | 22,660 (4.68) | 1 |
|  | Improved | 1,063,822 (68.71) | 118,979 (11.18) | 0.98 [0.972, 0.997] | 34,117 (3.21) | 0.94 [0.919, 0.958] |
| Household_Sooking | Low | 524,996 (33.95) | 75,128 (14.31) | 1 | 25,494 (4.86) | 1 |
|  | Medium | 289,928 (18.75) | 25,300 (8.73) | 1.01 [0.99, 1.04] | 6276 (2.16) | 0.90 [0.87, 0.94] |
|  | High | 731,319 (47.30) | 83,354 (11.40) | 1.03 [1.013, 1.043] | 24,946 (3.41) | 0.98 [0.91, 1.01] |
| Toilet | Unimproved ® | 1,013,764 (65.56) | 123,852 (12.22) | 1 | 21,715 (4.08) | 1 |
|  | Improved | 532,479 (34.44) | 59,930 (11.25) | 0.94 [0.92, 0.96] | 35,001 (3.45) | 0.86 [0.84, 0.88] |
| PM2.5 | below 5 μg/m3® | 23,331 (1.51) | 1923 (8.24) | 1 | 526 (2.25) | 1 |
|  | above 5 μg/m3 | 1,522,912 (98.49) | 181,859 (11.94) | 1.36 [1.23, 1.51] | 56,190 (3.69) | 1.77 [1.44, 2.17] |
| NO2 | below 10 µg/m3® | 1,448,705 (93.69) | 173,974 (12.01) | 1 | 54,084 (3.73) | 1 |
|  | above 10 µg/m3 | 97,538 (6.31) | 9808 (10.06) | 0.98 [0.95, 1.00] | 2632 (2.70) | 1.07 [1.02, 1.13] |
| Social GI |  |  |  | 0.98 [0.97, 0.98] |  | 0.97 [0.96, 0.97] |
| Political GI |  |  |  | 0.99 [0.99, 0.99] |  | 0.99 [0.99, 0.99] |
| Economic GI |  |  |  | 1.00 [1.00, 1.01] |  | 0.99 [0.99, 1.00] |
| Total Variance Partitions | | | | | | |
| District level variance |  |  |  | 0.09 [0.08, 0.11]*** |  | 0.17 [0.15, 0.19]*** |
| ICC District within country |  |  |  | 0.09 [0.07, 0.11]*** |  | 0.13 [0.10, 0.17]*** |
| Country level variance |  |  |  | 0.22 [0.15, 0.32]*** |  | 0.45 [0.22, 0.50]*** |
| ICC Country |  |  |  | 0.06 [0.04, 0.09]*** |  | 0.09 [0.06, 0.13]*** |

ICC: intra-level correlation coefficient, GI: Globalization index, CD, cesarean delivery; ®::reference, HHS: house hold size ***P-value < 0.001,**P-value < 0.01,*P-value < 0.05

The performances of the models were evaluated using different metrics and the result revealed that an increase in the number of protocols from K_2_ to K_5_ and then to K_10_, slightly improved the accuracy of the ML models. Moreover, the result revealed that all three training and testing data splits (80-20, 70-30, and 60-40%) with different protocols produced nearly equal performance metrics for all nine ML models. Overall, for all protocols (K_2_-K_10_) and all training/testing dataset ratios, the random forest (RF) had the highest performance in terms of AUC, accuracy, and other metrics. The RF model for K_2_ protocol with an 80/20 ratio achieved an AUC of 93.74% followed by the Bagged model (AUC=92.26%) performed well in all measures, indicating that they are effective in predicting the double and triple burdens of symptoms among under-five children across the LMICs (Fig. S8). However, the logistic regression (AUC=62.19%), Ridge (AUC=62.16%), LASSO (AUC=62.14), and decision tree (60.29%) models recorded the lowest metrics.


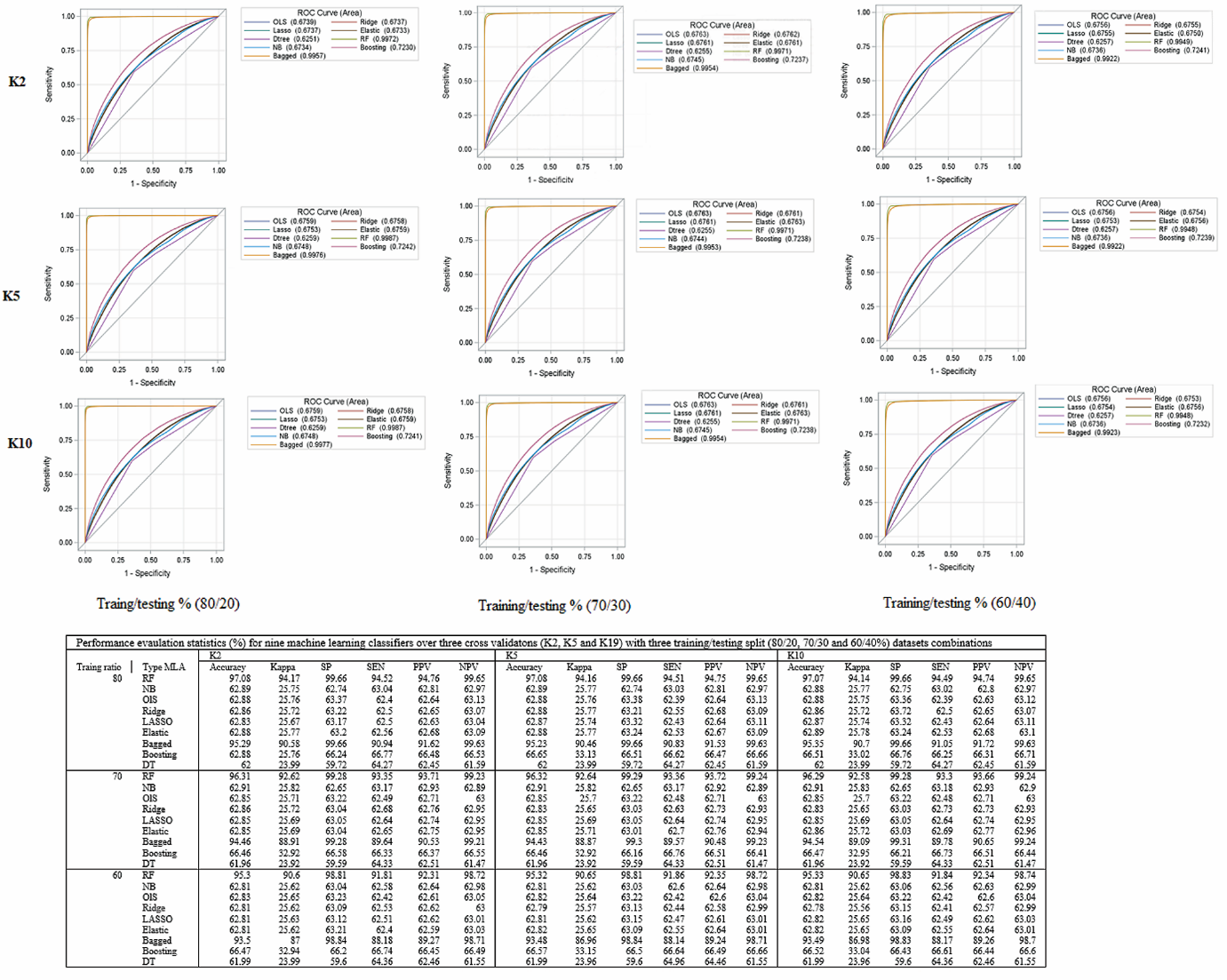


RF:random forest; NB: Naïve Baye;OLS: Ordinary logistic regression; DT: Decision trees; MLA: machine learning algorithms

Fig. S8: Performance comparison of machine learning algorithms to predict the DB symptoms with different metrics

References

1. Ahamad, M.G., F. Tanin, and N. Shrestha, *Household smoke-exposure risks associated with cooking fuels and cooking places in Tanzania: a cross-sectional analysis of demographic and health survey data.* International Journal of Environmental Research and Public Health, 2021. **18**(5): p. 2534.

2. Hammer, M.S., et al., *Global estimates and long-term trends of fine particulate matter concentrations (1998–2018).* Environmental Science & Technology, 2020. **54**(13): p. 7879-7890.

3. Gygli, S., et al., *The KOF globalisation index–revisited.* The Review of International Organizations, 2019. **14**: p. 543-574.

4. Croft, T.N., et al., *Guide to DHS statistics.* Rockville: ICF, 2018. **645**.

5. Vyas, S. and L. Kumaranayake, *Constructing socio-economic status indices: how to use principal components analysis.* Health policy and planning, 2006. **21**(6): p. 459-468.

6. Rutstein, S.O., *Steps to constructing the new DHS Wealth Index.* Rockville, MD: ICF International, 2015.
